# Approaches to motivate physicians and nurses in low- and middle-income countries: a systematic literature review

**DOI:** 10.1186/s12960-020-00522-7

**Published:** 2021-01-06

**Authors:** Jaya Gupta, Mariya C. Patwa, Angel Khuu, Andreea A. Creanga

**Affiliations:** 1 Johns Hopkins Bloomberg School of Public Health, 615 N. Wolfe Street, Baltimore, MD 21205 USA; 2 Johns Hopkins Bloomberg School of Public Health, Johns Hopkins School of Medicine, 615 N. Wolfe Street, Room E8646, Baltimore, MD 21205 USA

**Keywords:** Health worker motivation, Workforce, Supportive supervision, Systematic review, Low- and middle-income countries

## Abstract

Poor health worker motivation, and the resultant shortages and geographic imbalances of providers, impedes the provision of quality care in low- and middle-income countries (LMICs). This systematic review summarizes the evidence on interventions used to motivate health workers in LMICs. A standardized keyword search strategy was employed across five databases from September 2007 -September 2017. Studies had to meet the following criteria: original study; doctors and/or nurses as target population for intervention(s); work motivation as study outcome; study design with clearly defined comparison group; categorized as either a supervision, compensation, systems support, or lifelong learning intervention; and conducted in a LMIC setting. Two independent reviewers screened 3845 titles and abstracts and, subsequently, reviewed 269 full articles. Seven studies were retained from China (*n* = 1), Ghana (*n* = 2), Iran (*n* = 1), Mozambique (*n* = 1), and Zambia (*n* = 2). Study data and risk of bias were extracted using a standardized form. Though work motivation was the primary study outcome, four studies did not provide an outcome definition and five studies did not describe use of a theoretical framework in the ascertainment. Four studies used a randomized trial—group design, one used a non-randomized trial—group design, one used a cross-sectional design, and one used a pretest–posttest design. All three studies that found a significant positive effect on motivational outcomes had a supervision component. Of the three studies that found no effects on motivation, two were primarily compensation interventions and the third was a systems support intervention. One study found a significant negative effect of a compensation intervention on health worker motivation. In conducting this systematic review, we found there is limited evidence on successful interventions to motivate health workers in LMICs. True effects on select categories of health workers may have been obscured given that studies included health workers with a wide range of social and professional characteristics. Robust studies that use validated and culturally appropriate tools to assess worker motivation are greatly needed in the Sustainable Development Goals era.

## Background

Health workers, often the largest share of health budgets, are also responsible for managing other critical resources (e.g., vaccines, ventilators, and other essential drugs/commodities). To achieve the Sustainable Development Goals (SDGs), low- and middle-income-countries (LMICs) must contend with the following human-resource-for-health challenges: the shortage, maldistribution, poor-quality education, and limited competencies of health workers [1–3]. These factors contribute to three health workforce imbalances: numeric, geographic, and skill [4]. Respectively, these challenges are explained by the insufficient production of health workers, the poor retention of health workers in LMICs—especially in rural areas, and insufficient pre-service and in-service training of health workers [4]. These health workforce deficiencies further exacerbate health systems problems including access to care, equitable provision of care, and the quality of care [4]. Poor retention of health workers in LMICs, linked with corresponding numeric and geographic imbalances, is closely tied to poor motivation of health workers [5–9]. Motivation is defined by Franco et al. as “an individual’s degree of willingness to exert and maintain an effort towards organizational goals” [10]. Determinants of health worker motivation include individual-level (e.g., demographics, self-efficacy, etc.), organizational-level (e.g., resource availability, human resource management, etc.), and contextual (e.g., societal norms/values) factors [10, 11]. Migration push factors, stemming from discontentedness and dissatisfaction with work activities and the workplace, result in a concentration of providers in urban, compared to rural areas. This disparity is also contributed to by migration pull factors where health workers perceive improved prospects for promotion opportunities and enhanced living and working conditions in urban settings [12].

### Types of interventions to improve worker motivation

Human resource management (HRM) policies aim to address these conditions in rural and underserved areas by improving health worker retention and reducing maldistribution. Motivation of health workers is considered a core objective of such policies [4, 13]. Studies have found that there are a range of factors that may motivate health workers, such as job security, interesting work, a desire to gain respect, recognition, an adequate salary, and financial independence [14]. The World Health Organization (WHO) suggests that along the "working lifespan", there are three key intervention points: at a health worker’s entry into workforce, while the health worker is active in the workforce, and at the time of a health worker’s exit from the workforce. This review was limited to the study of interventions that target health workers *while they are active in the workforce*. Such interventions include supervision, compensation, systems support, and lifelong learning strategies [15].

Supervision interventions most commonly include supportive supervision, recognition, and career-development interventions. Supportive supervision is the process of assisting staff and employees in continuously improving their performance through non-authoritative and respectful methods. This approach facilitates open communication and seeks to develop a teamwork attitude towards problem solving [16]. One type of a supervision intervention, recognition interventions may take the form of either verbal commendation or receipt of an award from managers, supervisors, the community, or the government [8, 17]. Another possible supervision intervention, career development opportunities, are cited as one of the reasons health workers opt for urban as opposed to rural work sites, thereby acting as a pull-factor to bring health workers out of rural-posts where they are desperately needed [18]. Career-development interventions include promotion and specialization opportunities provided while in the workforce [8].

Compensation interventions are typically classified as financial incentives, such as provision of salary support, wage increases, bonuses, or performance-based financing; or fringe-benefit incentives, such as provision of transportation, food allowances, or housing [8, 11].

Systems support interventions aim at improving hospitals’ infrastructure and supply chains. Supply chain interventions relate to efforts made to address resource and personnel availability to ensure adequate stock and staff facilities. Upgrades to hospital infrastructure (i.e., physical construction)  have been previously linked with increased worker motivation [8, 19–21].

Lifelong learning interventions relate to opportunities for continuing education. In-service training is one such intervention that involves provision of continuous on-site training alongside peers during working hours.Studies have found that health workers take pride in furthering their education or abilities [8, 11, 22–24].

### Existing evidence on the effect of HRM on motivation

Chopra et al. (2008) conducted a review of systematic literature reviews to synthesize the evidence base for the effect of policy interventions (e.g., training, regulation, financing) on human resource outcomes. They identified 28 systematic reviews published between 1979 and 2006, of which only eight included studies conducted in LMICs. Moreover, while studies with outcomes such as supply, distribution, efficient use, and performance of health workers were all considered for inclusion in the large systematic review, those with work motivation outcomes were not [25]. It is possible that Chopra et al. did not retain those studies with motivation as a possible outcome due to the limited number of tools developed and validated for assessing health worker motivation. Indeed, to our knowledge, Willis-Shattuck et al. (2008) is the only study that sought to systematically review the effect of HRM interventions on health workers’ motivation from 1980 to 2007. The Willis-Shattuck review searched PubMed, ISI, Web of Science, Embase/Medline, as well as google scholar and the ‘Human Resources for Health’ online journal [8]. This systematic literature review of the effect of HRM interventions on health worker motivation in LMICs seeks to fill the gap in knowledge by summarizing the evidence produced in the subsequent 10 years, from 2007 to 2017. Our review diverges from the earlier Willis-Shattuck in that we incorporate an expanded search strategy with a robust librarian-generated keyword strategy and a cross-database extraction across additional databases. We also limited the outcome to health worker motivation (assessed quantitatively using a psychometric tool), specified a comparison-group study design, and limited the cadres of interest. Authors often draw causal relationships between HRM interventions and improved motivation in discussion sections—without sufficient evidence. By limiting the inclusion criteria to research with a control group (comparison or historical), we aim to identify the literature that has at least a plausibility level of inference [26].

## Methods

### Search strategy and study selection

A keyword search was conducted with assistance from a [Johns Hopkins]  University librarian. Five electronic databases were searched: PubMED, CINAHL Plus (includes "Human Resources for Health"), the World Health Organization Global Health Library (regional databases: LILACS, WPRIM, IMSEAR, IMEMR, AIM, WHOLIS), SCOPUS, and Embase for the period between September 1, 2007 to September 1, 2017; search restrictions included original research articles written in English. Search results were imported into Covidence (Covidence systematic review software, Veritas Health Innovation, Melbourne, Australia; www.covidence.org) and duplicate records were removed. Efforts were made to ensure that the search strategy did not miss eligible studies through a manual reference list search. An independent screening of article titles and abstracts was done by two reviewers (JG and MP) for potential inclusion in the review. Full-text articles were then obtained and reviewed in duplicate by two reviewers (JG and AK). Disagreement between reviewers was resolved through discussion and occasionally by involving a third reviewer (MP).

### Inclusion criteria

#### Types of participants

Studies that included facility-based interventions for nurses, nurse-midwives, or doctors who are facility-based were considered for inclusion in this review. Studies that were exclusively targeting other types of health personnel (e.g., dentists, lab-technicians, community health workers, etc.) were excluded from this review.

### Interventions to improve worker motivation

Given the wide scope of HRM interventions, this systematic review was limited to interventions at the *workforce stage,* including supervision, compensation, systems support, and lifelong learning interventions.

### Types of outcome measures

Since it is widely accepted that motivation is not directly observable or measurable, several psychometric tools have been developed in an effort to assess health worker motivation [27–31]. Only studies that present at least one outcome of motivation measured with a psychometric tool were included in this review.

### Types of studies

Only studies with either a pre–post or multi-arm comparison group were considered for inclusion. The following study designs were included:

Randomized trial—individual: at minimum two study arms with random assignment of study arm at individual level.

Randomized trial—group: at minimum two study arms with random assignment of study arm at group level (facility, district, etc.)

Non-randomized trial—individual: at minimum two study arms without random assignment of study arm at individual level.

Non-randomized trial—group: at minimum two study arms without random assignment of study arm at group level (facility, district, etc.)

Pretest–posttest: one study arm with one follow-up assessment period or assessment of outcome pre- and post-intervention implementation in same study population.

Time series: one study arm with multiple follow-up assessment periods or assessment of outcome pre-intervention and at several post-intervention periods.

Case–control group design: two study groups defined by level of the outcome with one group classified as cases and the other group classified as controls, where outcome levels are compared based on receipt of intervention.

Cross-sectional: two study arms with exposure and outcome determined at one time-point and comparison of outcomes in those who received intervention and those that did not.

### Data extraction

The full text data extraction was done in duplicate by two reviewers (JG, MP, or AK). The data extraction form developed for this study aimed to capture study characteristics (title, first author, study design, geographical location, study setting, participants’ characteristics, intervention details, data collection method, description of tool and how the outcome was measured, results, and study limitations). Measures of rigor and data quality were also extracted [32]. Assessment of the risk of bias in studies was conducted using the Cochrane Effective Practice and Organization of Care (EPOC) guidelines [33]. Specifically, as appropriate depending on study design, we assessed the random allocation of intervention, concealment of allocation, follow-up of respondents (where applicable) > 80% from baseline to endline, blind assessment of primary outcome(s), baseline measurement of outcome in both groups, baseline assessment of participants’ characteristics in both groups, reliability of the reported primary outcome(s), protection against study group contamination, and whether there was selective outcome reporting.

### Data synthesis

A narrative synthesis of studies was performed. Outcomes were reported as means, and a range of effects was provided where possible (either standard deviation, standard error, interquartile range, or 95% confidence interval). Plain text summaries were presented to contextualize results along with their statistical significance. Where studies included a baseline or comparison group, the means or proportions for both groups and/or the difference between groups were described as available. Where multiple points of follow-up were presented, the follow-up time closest to one year post-baseline was retained.

## Results

**Fig. 1:**
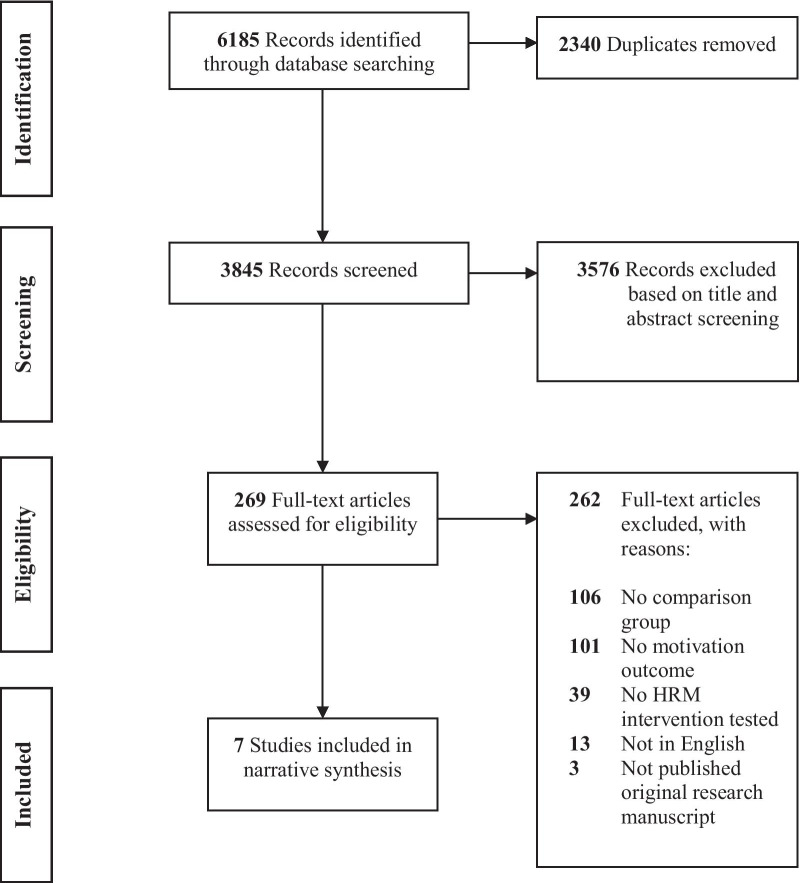
PRISMA flow diagram of study selection process

The database search identified 6,185 articles. After removing duplicates, 3,845 titles and abstracts were screened; 269 articles were selected for full-text review; and seven met all criteria for inclusion in our review (Fig. 1).

**Fig. 2:**
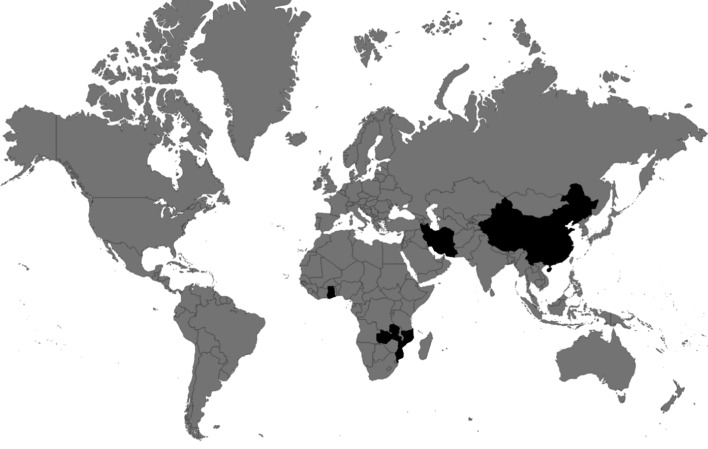
Countries in included studies

The seven articles presented results from studies in five countries: one in China [34], two in Ghana [35, 36], one in Iran [37], one in Mozambique [40], and two in Zambia [39, 40] (Fig. 2). Four of the studies used a randomized trial—group design [35, 37, 38, 40], randomizing the intervention at either the facility or district level; one study used a non-randomized trial—group design [36]; one used a cross-sectional design [39], and one used a pretest–posttest design [34]. While all studies included at least a nurse, doctor, and/or nurse-midwife cadre, all but one study [34] had heterogeneous target populations, thus including technicians, emergency personnel, pharmacists, and other types of health workers.

### Measurement of work motivation in included studies

Studies used a range of instruments to assess worker motivation. Tables 1 and 2 describe the instruments used by each study as well as the reported outcome(s). Aninanya et al. built on the work of Mutale et al. [31] and Mbindyo, Blaauw, et al. [30]; Hosseinabadi et al. [37] drew on the work of Mohsenpour et al. [29] and Jafariayan [28]; Liu et al. [34] used the 38-item Chinese version of the Practice Environment Scale (CPPE-38); Shen et al. [40] used the Weiss et al. (1967) Minnesota Satisfaction Questionnaire and Spector et al. (1985) Job Satisfaction Survey; Vermandere et al. [38] also built on work done by Mutale et al. [31]. Three studies defined motivation [35, 36, 40] using the definition provided by Franco et al. [10]. Two studies used the Herzberg two-factor theory of motivation, which dichotomizes motivation into intrinsic and extrinsic motivation [37, 39].

**Table 1:** Description of study design, interventions, and methods of outcome ascertainment for studies in the systematic literature review

Author	Country	Study design	Respondents	Intervention type	Description of intervention	Random allocation	Motivation definition provided	Instrument informed by motivation theory	Items/instrument used
Alhassan et al. 2016	Ghana	Randomized trial—group	Heterogeneous: clinical and non-clinical staff	Supervision	The Systematic Community Engagement (SCE) intervention included a structured step-by-step and cyclical process of facilitators engaging community groups/associations to assess health service quality at their nearest health facility. Facilitators provided feedback to facility heads, gaps in quality were identified, and action plans were developed to address gaps. Facilitators followed up with service providers to monitor progress on action plan.^a^ Sixteen facilities were assigned to intervention, and sixteen served as controls.	Y	Y^2^	Not described	Not described
Aninanya et al. 2016	Ghana	Non-randomized trial—group	Heterogeneous: community health nurses; midwives; other-medical assistants and public health nurses	Compensation; Supervision	Performance-based intervention (PBI) with financial and non-financial awards provided to the best-performing health workers at biannual ceremonies. Awards included monthly allowances (~ US$20), small appliances (e.g., refrigerators, televisions, microwaves) or certificates of recognition. Six facilities were allocated to the intervention group receiving PBI, and six facilities served as controls.	N	Y^2^	Not described	Mutale et al. 2013 & Mbindyo et al. 2009
Carasso et al. 2012	Zambia	Cross-sectional	Heterogeneous: nurses; doctors; midwives; pharmacy dispensers; classified daily employees	Compensation	Intervention facilities abolished a user fee, which authors theorize results in a loss of financial incentives to health worker and concurrent increase in utilization. The control group retained the user fee, theoretically improving financial incentives to health workers. The six facilities that continued to collect user fees were the intervention, and the fourteen facilities where user fees were abolished were the control. As the reverse policy would serve as an incentive to providers, we consider the retention of user fees to be the intervention.	N	N	Y—Hertzberg two-factor motivation theory	Not described
Hosseinabadi et al. 2013	Iran	Randomized trial—group	Heterogeneous: emergency medical service personnel (including emergency medical technicians, nurses, operating room attendants, and anesthetic technicians)	Supervision	Quality circles implemented as a participatory management technique to offer assistance to health workers dealing with work-related problems and led by a supervisor. The goal was to resolve work-related problems, improve performance and motivate employees. One facility served as the intervention facility and one facility served as the control.	Y	N	Y—Hertzberg two-factor motivation theory	Mohsenpour et al. 2002; Jafariayan, 2007
Liu et al. 2017	China	Pretest–posttest	Homogenous: registered nurses; registered professional nurses; nurse practitioners; assistant head nurse	Supervision; Compensation; Lifelong learning	A web-based communication platform used to document comments from nurses and psychological forum held twice a year to discuss these; 2nd component to provide continuing education and certificates for nurses; 3rd component to offer spiritual rewards to encourage internal motivation of nurses. Salary and benefits were raised based on the performance appraisals. Study was conducted in one facility with historical control.	N	N	Not described	38-item Chinese version of the Practice Environment Scale (CPPE-38)
Shen et al. 2017	Zambia	Randomized trial—group	Heterogeneous: district community medical officer; clinical officer; registered nurse; enrolled midwife; enrolled nurse; environmental health technician; classified daily employee, lab technician, other (administrators, human resource officers)	Compensation	Performance-based financing (PBF) was one intervention group, with financing linked to performance; Enhanced financing (EF) was the first control group, where the same amount of financing was given but not linked to performance; the pure control facilities were the third group where no financing was given Due to bottlenecks- the EF group only received about 56% of the financing amount as the PBF group- and therefore was dissimilar. Thirty districts were triplet-matched on key health systems and outcome indicators and randomly allocated to one of three arms, ten to PBF, ten to Control-Enhanced Financing, ten to a pure control.	Y	Y^2^	Not described	Weiss et al. 1967: Minnesota Satisfaction Questionnaire Spector et al. 1985: Job Satisfaction Survey
Vermandere et al. 2017	Mozambique	Randomized trial—group	Heterogeneous: health care providers	Systems support	The intervention consisted of ten monthly audits in 15 facilities to examine stock cards and stock-counts of six contraceptives. The first intervention group received only a monthly evaluation report, reflecting the quality of their supply management. The second intervention group received the monthly evaluation report as well as material incentives conditional on facility performance (note: incentives at facility, not individual, level). The third group served as a control group. Fifteen health facilities in Maputo Province, Mozambique, were divided into 3 groups of five facilities: intervention group (monthly evaluation report), a second intervention group (monthly evaluation report and incentives), and finally a third group (control).	Y	N	Not described	Mutale et al. 2013

^a^Alhassanet al. (2013)

^**b**^Definition provided refers to "an individual’s degree of willingness to exert and maintain an effort towards organizational goals" (Franco et al. 2002)

**Table 2:** Description of study characteristics, reported outcomes, and narrative results for all seven studies retained from the systematic review

Author	Intervention group	Instrument details	Sampling	Groups	Reported outcome	Results	Significant findings	Narrative description of results
Alhassan et al. (2016)	Supervision	19 items 4-point Likert scale ranging from (1) "Very Disappointing" to (4) "Very Satisfactory" Reliability: Cronbach's alpha: >0.7	234 participants at endline (Intervention = 103, Control = 131)	Group 1 (intervention): 32 facilities received SCE intervention Group 2 (control): 32 control facilities	Motivation Factor 1: Physical work environment and resource availability	Difference between Intervention and Control at Baseline: mean= −0.01 (SE= 0.01) Difference between Intervention and Control at Follow-up (2 years later): mean= 0.10 (SE=0.08) Difference in Difference: mean= 0.11 (SE=0.12)	NS	Difference-in-difference analyses of the levels of staff motivation from 2012 to 2014 found that SCE-receiving facilities rated motivation proxies (career prospects, perceived workload, and overall motivation) higher than non-SCE facilities. (p<0.0001) The association between SCE and financial incentives and physical work environment was low or negative.
					Motivation Factor 2: Financial and extrinsic incentives^1^	Difference between Intervention and Control at Baseline: mean= −0.19 (SE= 0.01) Difference between Intervention and Control at Follow-up (2 years later): mean= −0.09 (SE=0.08) Difference in Difference: mean = −0.28 (SE = 0.12)	NS	
					Motivation Factor 3: Intrinsic incentives^b^	Difference between Intervention and Control at Baseline: mean= 0.22 (SE= 0.04) Difference between Intervention and Control at Follow-up (2 years later): mean= 0.44 (SE=0.07) Difference in Difference: mean = 0.22 (SE = 0.08)	NS	
					Motivation Factor 4: Career prospects and opportunities for further education	Difference between Intervention and Control at Baseline: mean= 0.68 (SE= 0.11) Difference between Intervention and Control at Follow-up (2 years later): mean= 0.79 (SE=0.10) Difference in Difference: mean=0.11 (SE=0.17)	+	
					Motivation Factor 5: Perceived workload and staff availability	Difference between Intervention and Control at Baseline: mean= 0.51 (SE= 0.11) Difference between Intervention and Control at Follow-up (2 years later): mean= 0.51 (SE=0.08) Difference in Difference: mean=0.00 (SE=0.14)	+	
					Overall motivation^e^	Difference between Intervention and Control at Baseline: mean= 0.24 (SE= 0.04) Difference between Intervention and Control at Follow-up (2 years later):mean= 0.32 (SE=0.06) Difference in Difference: mean=0.08 (SE=0.07)	+	
Aninanya et al. 2016	Compensation; Supervision	14 items4-point Likert scale ranging from (1) "Strongly agree" to (4) "Strongly disagree" Reliability: Cronbach's Alpha: 0.7041	50 participants at endline (Intervention = 25; Control = 25)	Group 1 (intervention): 6 facilities allocated as intervention-receiving PBI Group 2 (control): 6 facilities allocated as comparison – did not receive PBI	Motivational construct: Intrinsic Motivation	Difference between Intervention and Control at Baseline: Score= -0.25 Difference between Intervention and Control at Endline (3 years later):Score= 0.20 Difference-in-Difference:Score= 0.00	NS	Overall motivation was slightly higher in comparison compared to intervention group. Overall motivation increased in intervention group, but remained constant in comparison group over time. Difference-in-difference was small. All changes in motivation were not statistically significant.
					Overall motivation^5^	Difference between Intervention and Control at Baseline: Score= -0.11 Difference between Intervention and Control at Endline (3 years later): Score= 0.08 Difference-in-Difference:Score= 0.01	NS	
Carasso et al. 2012	Compensation	3 items 5-point Likert scale ranging from (1) "Very satisfied" to (5) "Very dissatisfied" Reliability: Cronbach's Alpha: 0.67	90 participants at endline (Intervention = 33; Control = 57)	Group 1 (intervention): 14 facilities where user fees were removed- resulting in lower worker financing Group 2 (control): 6 facilities where user fees were still charged-resulting in higher worker financing	Extrinsic motivation	Mean motivation at endline for Intervention group: Mean= -0.55 Mean motivation at endline for Control group: Mean= 0.33	-	Staff in intervention group (with poorer health financing) reported higher extrinsic motivation than those in the control group. This finding was statistically significant (p<0.05).
Hosseinabadi et al. 2013	Supervision	17 items 5-point Likert scale Range not described Reliability: Stated as reliable; not described in paper	40 participants at endline (Intervention = 24; Control = 16)	Group 1 (intervention): 1 facility received quality circles Group 2 (control): 1 facility did not receive quality circles	Motivational Factors	Mean motivation at baseline for Intervention Group: mean= 35.25 (SD= 8.38) Mean motivation at endline (3 months) for Intervention Group: mean=43.50 (SD=7.63) Mean motivation at baseline for Control Group: mean=36.68 (SD= 8.51) Mean motivation at endline (3 months) for Control Group: mean= 34.25 (SD= 7.93)	+	There were no statistically significant differences in mean motivational scores between groups at baseline. At endline, motivation was significantly higher in intervention compared to control group.
Liu et al. 2017	Supervision; Compensation; Lifelong learning	10 items 4-point Likert scale ranging from (1)"Strongly agree" to (4) "Strongly disagree" Reliability: not described	1050 participants at baseline 920 participants at endline	Group 1 (baseline): 1 facility receiving life-long learning and compensation intervention Group 1 (endline): historical control – same facility did not receive life-long learning and compensation intervention	Internal work Motivation	Score at baseline:Mean= 3.15 (SD= 0.40) Score at endline: mean= 3.22 (SD= 0.64) Difference in score from baseline to follow-up (2-years later) not reported. Calculated by authors to be: mean= 0.07	+	Post intervention, there was a statistically significant positive change in internal work motivation over time (p<0.05). Internal motivation differed by the assigned work unit- with those from the operating room and post-anesthesia care unit demonstrating the lowest scores of internal work motivation.
Shen et al. 2017	Compensation	39-items 5-point Likert scale ranging from (1) "Very dissatisfied" to (5) "Very satisfied" Reliability: not described	326 participants at baseline (Intervention = 147, Control 1 = 87, Control 2 = 92) 357 participants at endline (Intervention: 166, Control 1 = 92, Control 2 = 99)	Group 1 (intervention): 10 districts received PBF Group 2 (control 1): 10 districts received EF Group 3: (control 2): 10 districts received no PBF and no EF	Motivational Construct 1: Teamwork	Pairwise regression results: PBF v. EF: β=0.39 SE=3.13 PBF v. Control: β=0.93 SE=1.43 EF v. Control: β=1.62 SE=3.51	NS	There was no statistically significant difference in any of the motivational outcomes between groups.
					Motivational Construct 2: Autonomy	Pairwise regression results: PBF v. EF: β=0.82 SE=4.31 PBF v. Control: β=1.31 SE=1.77 EF v. Control: β=1.30 SE=4.49	NS	
					Motivational Construct 3: Recognition	Pairwise regression results: PBF v. EF: β=0.38 SE=3.28 PBF v. Control: β=-0.84 SE=1.33 EF v. Control: β=-0.89 SE=2.85	NS	
					Motivational Construct 4: Change	Pairwise regression results: PBF v. EF: β=-2.10 SE=2.66 PBF v. Control: β=1.03 SE=1.24 EF v. Control: β=3.83 SE=2.64	NS	
					Motivational Construct 5: Self-concept	Pairwise regression results: PBF v. EF: β=-0.73 SE=1.87 PBF v. Control: β=0.77 SE=1.08 EF v. Control: β=2.21 SE=2.36	NS	
					Motivational Construct 6: Work environment	Pairwise regression results: PBF v. EF: β=-1.79 SE=2.60 PBF v. Control: β=1.26 SE=1.26 EF v. Control: β=4.31 SE=3.03	NS	
					Motivational Construct 7: Leadership	Pairwise regression results: PBF v. EF: β=-3.08 SE=4.89 PBF v. Control: β=1.21 SE=2.61 EF v. Control: β=5.55 SE=5.15	NS	
					Motivational Construct 8: Well-being	Pairwise regression results: PBF v. EF: β=1.10 SE=2.98 PBF v. Control: β=2.42^a^ SE=1.24 EF v. Control: β=3.93 SE=2.50	NS	
Vermandere et al. 2017	Systems support	23 items 5-point Likert scale ranging from (1) "Strongly agree" to (5) "Strongly disagree" Reliability: not described	55 participants at baseline (Group 1 = 17, Group 2 = 16, Group 3 = 22) 40 participants at 1^st^ follow-up (Group 1 = 12, Group 2 = 12, Group 3 = 16) 39 participants at 2^nd^ follow-up (Group 1 = 10, Group 2 = 12, Group 3 = 17)	Group 1 (intervention 1): 5 facilities receiving monthly evaluation Group 2 (intervention 2): 5 facilities receiving monthly evaluation + financial incentives Group 3 (control): 5 facilities receiving not receiving facility audits	Overall motivation^e^	Motivation at Baseline (Group 1): median=88.5 IQR=87–92 Motivation at Follow-up 2 (1 year later) (Group 1): median=90 IQR=88–90 Motivation at Baseline (Group 2): median=93 IQR=86.5–95 Motivation at Follow-up 2 (1 year later) (Group 2): median=87 IQR=83–90 Motivation at Baseline (Group 3): median=84.5 IQR=79–93 Motivation at Follow-up 2 (1 year later) (Group 3): median=87 IQR=83–90 None of the differences between 2nd follow-up and baseline were reported but *p* values presented were all non-significant	NS	There was no statistically significant difference in motivation between intervention group and comparison group at baseline, endline, or over time

Significant findings: + is positive,—is negative, *NS* non-significant findings at 95%

^a^Financial and material work conditions of a job (e.g., salary increment, promotion, accommodation, etc.)

^b^Inner joy and satisfaction derived from a job (e.g., societal recognition and respect; appreciation shown by clients, etc.)

^c^As authors only provided a pooled sample size from baseline and follow-up surveys, reported sample size is the same at baseline and follow-up

^d^Baseline or control performance is the pre-intervention performance in the intervention arm, or if not measured, the end performance in the control arm

^e^Composite score of multiple constructs

Across the seven studies, the number of items used to capture the motivational outcome ranged from three to thirty-nine. All studies used psychometric instruments on a Likert scale, with four studies opting for a 5-point scale and three studies opting for a 4-point scale. Three scales asked about degree of "agreement" with statements provided; three scales asked about the degree of "satisfaction" with statements provided; and one study did not specify the Likert scale options. Alhassan et al. [35] and Aninanya et al. [36] used work motivation tools with good internal reliability (Cronbach's alpha scores > = 0.7). For the other five studies, worker motivation instrument reliability was either not reported or < 0.7. No study provided reliability measures (e.g., Kappa measures) for inter-rater reliability of the outcome.

Study interventions broadly matched one or more of the four HRM categories of supervision, compensation, systems support, and lifelong learning. Four studies included an intervention that incorporated a component of supervision (Table 2) [34–37]. Alhassan et al. [35] assessed a Systematic Community Engagement (SCE) intervention in Ghana that consisted of community group feedback provided in facilities, with facilitators communicating this feedback to service providers and monitoring subsequent progress made to address it using an outlined action plan [36]. Aninanya et al. assessed another intervention in Ghana that offered both financial and non-financial awards to providers based on their job performance. This intervention also had a supervision component entailing receipt of certificates of recognition and award ceremonies for best-performing providers [36]. Hosseinabadi et al. (2013) assessed a supervision intervention in Iran, which entailed use of facility quality circles with emergency medical personnel (medics, nurses, etc.), where health workers were provided a space to discuss challenges with guidance from a supervisor [37]. Liu et al. assessed an intervention that was primarily related to financial incentives, though the intervention also included components of supervision where nurse employees were granted a space to discuss work-related challenges as well as given opportunities for recognition [34].

Four studies included a compensation component (Table 2) [34, 36, 39, 40]. Aninanya et al. assessed the implementation of performance-based interventions (PBI) in Ghana, where monthly allowances (~ $20) and small appliances (e.g., refrigerators, televisions, microwaves) were provided as incentives for best-performing health workers [36]. Carasso et al. assessed the effect of a policy-change in Zambia–the removal of user fees in some facilities ("non-charging" or financial incentive reductions for providers) compared to the continuation of regular user fees in other facilities ("charging" or financial incentives increased for providers due to increased revenue) [39]. Liu et al. assessed an intervention in China where provider salary and benefits were raised based on performance appraisals and also included a component of life-long learning with continuing education provided to nurses as part of the intervention [34]. Shen et al. assessed an intervention in Zambia where three groups of districts were compared: a performance-based financing (PBF) group; a group receiving Enhanced Financing (EF)-funding in the same amount as the PBF group, but not tied to performance; and a group that did not receive additional financial compensation. Of note, the EF group only received about 56% of intended funding due to issues with administrative bottlenecks and financing processes [40]. Vermandere et al. was the only study to assess the effect of a systems support intervention on health worker motivation. The intervention was implemented in Mozambique and consisted of facility-audits for contraceptive stock-outs used to improve the quality of supply management [38].

### Effect of interventions in studies included in the review

Three studies found a significant effect of the interventions implemented on health worker motivation [34, 35, 37]. Alhassan et al. found that providers in facilities receiving the SCE intervention rated motivation proxies of career prospects, perceived workload as well as overall work motivation significantly higher compared to providers in control facilities [35]. Hosseinabadi et al. found that providers in intervention facilities (i.e., those facilities receiving supportive supervision in the form of facility quality circles) reported significantly higher mean motivation compared to those in the control group at endline [37]. Liu et al. found a statistically significant positive change in internal work motivation among nursing cadres post-implementation of the intervention—which included a complaint forum, a continuing education platform, and financial performance-based rewards [34].

Three studies found no effect of interventions studied on motivational outcomes [36, 38, 40]. Aninanya et al. found no significant difference in intrinsic or overall motivation of nurses, midwives, and medical assistants in PBI/award receiving facilities versus those in comparison facilities [36]. Shen et al. did not find any significant difference in work motivation constructs between providers in intervention and control groups following performance-based financing implemented in the intervention group [40]. Vermandere et al. found that neither intervention group (facility-audit only group or facility audit/financial-incentive group) reported significantly different overall motivation than the control group at endline [38].

In one study, Carasso et al. found a negative effect of financial incentives (resulting from retention of user fees) on health worker motivation compared to a control group where there reduced financial incentives (due to abolition of user fees). In facilities where providers’ continued to receive financial incentives resulting from user fees, extrinsic motivation was significantly lower compared to providers in facilities where user fees were eliminated [39].

### Risk of bias in included studies

Four studies [34, 36–39, 41] described randomly allocating the group to receive the intervention or not, which implies a relatively low risk of selection bias in these studies compared to the other three (Table 3). Concealment of study group allocation to either participants or assessors to prevent performance or detection bias was not done in any study. Concealment of human resource interventions was not possible given that they are typically implemented at the facility-level. Out of the six studies where follow-up was possible, three studies did not meet EPOC standards of < 20% loss to follow-up after randomization outlined by EPOC [35, 37, 38]. In one study, follow-up was > 80% [34]; and in the other two studies, follow-up was not described [36, 40]. None of the studies had blinded assessment of the primary outcome as, due to the nature of health systems interventions, blinding is typically not possible. Of the five studies where there was follow-up and a comparison group, two studies [36, 37] included baseline measurement of outcome (i.e., work motivation) and differences between groups as per EPOC standards; three did not [35, 38, 40]. Only one study provided baseline demographic characteristics by intervention group and described significant differences as per EPOC standards [37]. Studies were typically protected from contamination given that interventions were allocated at the facility-level as opposed to the individual level. However, in some instances, authors reported concern about contamination due to timing of external interventions or due to district pairing [36, 40]. In all studies included in our review except for one [39], there was no selective outcome reporting, given that positive, null, and negative findings were all reported. Additional risk of bias assessment details are provided in Table 3. All studies retained introduced some risk of bias and interpretation of results should be done with caution and within the context of these study limitations.

**Table 3:** Risk of bias in included studies

Risk of bias	Assessment of bias in studies included in the lit review	General description
Random allocation of intervention	Yes (1–4)	Four studies randomized the intervention at a facility/district level, whereas the other three studies did not randomize allocation of intervention
	No (5–7)	
	Not clear	
	Not applicable	
Concealment of allocation	Yes	No studies concealed assignment. This is expected given the nature of health systems interventions
	No (1–7)	
	Not clear	
Follow-up of professionals	Yes (7)	Out of the six studies where follow-up was possible, four studies did not have the adequate 80–100% follow-up after randomization outlined by EPOC. In the other two studies, follow-up was not described
	No (1,3,4)	
	Not clear (2,5)	
	Not applicable (6)	
Blinded assessment of primary outcome(s)	Yes	None of the studies had blinded assessment of the primary outcome, as it is not possible given the nature of health systems interventions
	No (1–7)	
	Not clear	
	Not applicable	
Baseline measurement of outcomes in both groups	Yes (4,5)	Two studies included baseline measurement of outcome and differences between groups, three did not, and in two cases it was not applicable
	No (1–3)	
	Not clear	
	Not applicable (6,7)	
Baseline measurement of characteristics in both groups	Yes (4)	Most studies had unclear, or did not describe, baseline measurements of characteristics in both groups, with some reporting pooled numbers or not providing differences. One article did
	No (1,3)	
	Not clear (2,5)	
	Not applicable (6,7)	
Reliable primary outcome measure(s)	Yes	No studies presented an inter-rater reliability measure
	No (1–7)	
	Not clear	
	Not applicable	
Protection against contamination	Yes (1,3,4,6)	Almost all studies had an element of randomization, with randomization done at a facility or district level. These processes help reduce risk contamination. In the remaining two cases with comparison groups, contamination may have been compromised due to other external programs and district pairing
	No	
	Not clear (2,5)	
	Not applicable (7)	
No selective outcome reporting	Yes (1–5, 7)	Most studies reported both significant and non-significant findings. In one study, it was not clear whether there was selective reporting
	No	
	Not clear (6)	
	Not applicable	

(1) Alhassan et al. (2016), (2) Shen et al. (2017), (3) Vermandere et al. (2017), (4) Hosseinabadi et al. (2013), (5) Aninanya et al. (2016), (6) Carasso et al. (2012), (7) Liu et al. (2017)

## Discussion

Despite our use of a conservative inclusion criteria for this systematic literature review, only seven studies have aimed to document the effect of key HRM interventions on health worker motivation using quantitative methods and an appropriately chosen comparison group, either historical or control. Chopra et al. found that overall few systematic reviews have been conducted to understand the effect of human resource policy options in low- and middle-income settings. The majority of those that do exist focus on lay health workers rather than facility-based clinical providers [26]. Past systematic reviews that have reviewed the effect of interventions to retain or reduce emigration of health workers retained zero and one study, respectively [41, 42].

Interventions assessed in this review were complex and, by and large, included multiple components. All three studies that examined interventions with a supervision component demonstrated favorable effects on health worker motivational outcomes, one study found that higher provider financing had a negative effect on motivation, and the other three studies found null effects of interventions on motivation. Results from these seven studies provide limited evidence of promising or successful interventions to motivate health workers in LMICs, and they are considerably hindered by the heterogeneity of the settings, study populations, the different methods of outcome ascertainment and other methodological concerns.

All interventions that demonstrated some effect on motivation had a supervision component. This finding is consistent with related literature that found poor supervision is a strong predictor of health worker intention to leave and poor job satisfaction [43, 44]. Additionally, prior qualitative studies have detailed health worker frustration with the provision of limited supervision (no written or oral feedback) as well as the negative tone of feedback [19]. Supervision is theorized to improve worker motivation through a greater connection between individual and health system, improving individuals' orientation to organizational values [45, 46]. Feedback on job performance may also improve an individual’s sense of competency, which self-determination theorists posit may enhance self-motivation [47].

We also found that the retention of user fees resulting in improved health financing was negatively associated with extrinsic motivation. The literature to date examining the effect of health financing on motivation has been focused on the potential role of performance-based financing to undermine intrinsic motivation [48–50]. Carasso et al. supplemented their negative quantitative findings with qualitative inquiry, which provided some insight as to why improved motivation was higher among providers receiving lower financial incentives. Among qualitative findings, providers remarked upon how the abolition of user fees resulted in the reduction of income and allowances and an increase in workload. However, many described an improved sense of personal satisfaction with reduction of user fees as they were no longer limited in their ability to provide care for the poorest within the community. Some providers also noted paying for their patients' user fees after seeing their medical condition due to a sense of duty as a provider. Taken together, these valuable qualitative findings depict a perceived tradeoff between poorer remuneration and a greater sense of doing rewarding work [39].

Given that this systematic review was limited to studies published in the peer-reviewed literature and did not assess grey literature, there is risk of publication bias in our review. As there is no standard practice to assess motivation, the outcome of interest was oftentimes assessed using different tools. Thus, a meta-analysis of the seven studies was not possible. Yet, we captured and documented similarities and differences in the methods of ascertainment of outcomes to inform future research efforts. Another notable limitation of our systematic reviews is that the majority of the studies included in our review are of relatively poor methodological quality. HRM interventions may not alone explain differences in individual motivation, and efforts were made to reduce potential for residual confounding by limiting the inclusion criteria to study designs with a control group and/or randomized control trial design.

A number of best practice considerations for future interventions and research to assess the effect of interventions on motivation come out of our review: (i) use a reliable, valid, and culturally appropriate and theory-based work motivation assessment tool; (ii) use homogeneous settings and study populations in all of the groups being compared; (iii) improve the rigor of the study design in order to better establish causal relationships. Ideally, to develop a strong body of evidence with regard to how to motivate health workers, consensus should be reached in the scientific community regarding the best health worker motivation tool or menu of tools to be employed. Three of the studies in our review aimed to operationalize the Franco et al. (2002) definition of motivation [10]. However, there is a large body of literature assessing job-satisfaction rather than work motivation—the two constructs, while related, are not equivalent. As consistency in employing work motivation as a latent construct in research studies improves, we will be closer to understanding what interventions work to motivate health workers. Further, it is known that motivation is likely constructed differently for health workers based on their demographic characteristics (e.g., age and gender) as well as employment characteristics (status—permanent or contractual, cadre—doctor or nurse, etc.). As such, future studies should conduct studies on homogenous populations or disaggregate outcomes by groups [51, 52].

Finally, the use of cross-sectional surveys and non-probabilistic sampling make it impossible to draw causal inferences. Small sample sizes or units of randomization further limit the ability to draw causal inferences. In a 2010 WHO report, outlining guidelines for the rural retention of health workers, experts noted that the quality of evidence—by clinical appraisal research standards—was low or very low for most recommendations [53]. As in this review, the report advocates for the improved rigor of effectiveness research study designs, while also acknowledging the benefits of existing evidence. Though in this systematic review, efforts were made to collate the evidence at a minimum plausibility level of inference, other study designs are available in this field that are relevant and valid. Qualitative research, in particular, is critical to unpacking the “why” and “how” of intervention effectiveness. Yet, more robust, randomized study designs are also needed to evaluate HRM interventions.

## Conclusion

In conclusion, there remains a dearth of evidence about the effect of interventions to improve health worker motivation in LMICs, which in turn, can affect entire health systems by improving access to, quality of, and equity in health care. Existing studies have many limitations, lacking consistent definitions of work motivation, a rigorous study methodology, and specificity as to who received the intervention, thus potentially obscuring the true effects of interventions on health worker motivation. An evidence base for methods to motivate health workers is required to ensure health workforce shortages can be remediated in LMICs through appropriate human resource management interventions.

## Data Availability

Not applicable.

## Abbreviations

AIM: African Index Medicus
EF: Enhanced Financing
EPOC: (Cochrane) Effective Practice and Organisation of Care
HRM: Human Resource Management
IMEMR: Index Medicus for the Eastern Mediterranean Region
IMSEAR: Index Medicus for South-East Region
LILACs: Latin American & Caribbean Health Sciences Literature
LMICs: Low- and Middle-Income Countries
PBF: Performance-based financing
SDGs: Sustainable Development Goals
SCE: Systematic Community Engagement
UNICEF: United Nations International Children's Fund
WHO: World Health Organization
WHOLIS: World Health Library Database
WPRIM: Western Pacific Region Index Medicus
